# The accuracy of electromagnetic navigation bronchoscopy compared to fluoroscopy in navigation of transbronchial lung cryobiopsy in patients with interstitial lung disease

**DOI:** 10.1186/s12890-024-02925-x

**Published:** 2024-03-04

**Authors:** Shiyao Wang, Jinmi Yang, Sa Luo, Jing Geng, Yanhong Ren, Ling Zhao, Min Liu, Dan Wang, Ying Li, Zheng Tian, Wei Liu, Guowu Zhou, Huaping Dai

**Affiliations:** 1 National Center for Respiratory Medicine; State Key Laboratory of Respiratory Health and Multimorbidity; National Clinical Research Center for Respiratory Diseases; Institute of Respiratory Medicine, Chinese Academy of Medical Sciences, Department of Pulmonary and Critical Care Medicine, Center of Respiratory Medicine, China-Japan Friendship Hospital, #2 Yinghuayuan East Street, 100029 Beijing, China; 2 Department of Pulmonary and Critical Care Medicine, Inner Mongolia Autonomous Region People’s Hospital, Hohhot, 010017 Inner Mongolia Autonomous Region China; 3 Department of Pathology, China-Japan Friendship Hospital, 100029 Beijing, China; 4 Department of Radiology, China-Japan Friendship Hospital, 100029 Beijing, China

**Keywords:** Transbronchial lung cryobiopsy, Electromagnetic navigation bronchoscopy, Fluoroscopy, Interstitial lung disease

## Abstract

**Background:**

Safely implementing transbronchial lung cryobiopsy (TBLC) in patients with interstitial lung disease (ILD) requires accurate navigation. Traditional fluoroscopy falls short in reducing the risk of post-procedure pneumothorax. The potential of electromagnetic navigation bronchoscopy (ENB) as a more precise navigation method warrants further exploration.

**Methods:**

A prospective cohort study was conducted on ILD patients undergoing TBLC. Patients were assigned either fluoroscopy or ENB for cryoprobe positioning. Navigation accuracy was evaluated using cone beam computed tomography (CBCT) images as the standard. Safety and diagnostic yield were also observed.

**Results:**

Seventeen patients underwent TBLC, with 10 guided by fluoroscopy and seven by ENB. Fluoroscopy-guided cryoprobe navigation required more adjustments [9/15 (60%) v.s. 1/9 (11%), *p* = 0.018] for subsequent TBLC compared to ENB, as confirmed by CBCT images. Clinical characteristics, post-procedure complications, and biopsy specimen size showed no significant differences between the groups. Fourteen patients obtained a pathological diagnosis, and 15 received a multidisciplinary discussion (MDD) diagnosis. In the fluoroscopy group, three patients failed to obtain a pathological diagnosis, and two failed to obtain an MDD diagnosis.

**Conclusions:**

ENB demonstrates significantly superior accuracy in TBLC navigation compared to traditional fluoroscopy when CBCT images are used as a reference. Further studies are necessary to determine the value of ENB in TBLC navigation for ILD patients.

## Introduction

Transbronchial lung cryobiopsy (TBLC) is increasingly applied in the diagnosis of interstitial lung disease (ILD), and it has been considered an alternative to surgical lung biopsy (SLB), which was once considered the gold standard for the pathological diagnosis of ILD [1]. The safety of TBLC has been proved in many studies, but its main complications remain airway bleeding and pneumothorax [2–5]. Therefore, navigation is required during the TBLC procedure to determine whether the cryoprobe is positioned properly to avoid proximity to the great vessels and pleura. Among various navigation methods, fluoroscopy is the earliest and most commonly used [6, 7]. The application of fluoroscopy during TBLC is also recommended in the CHEST Guideline and Expert Panel Report [8]. However, previous studies have shown that fluoroscopy navigated TBLC has a high incidence of pneumothorax complications [9].

Therefore, other navigation methods that can reduce the risk of complications of TBLC are gradually being explored, among which cone beam computed tomography (CBCT)-navigated TBLC can significantly reduce the incidence of post-procedure pneumothorax and airway bleeding [3, 10, 11]. However, the scarcity of CBCT equipment and radiation exposure limits its wide application in TBLC navigation. Electromagnetic Navigation Bronchoscopy (ENB) is a relatively new technique for bronchoscopy navigation. It utilizes reconstructed virtual three-dimensional images of the tracheobronchial tree derived from high-resolution chest imaging. The process involves setting navigation goals and planning paths within a specific system, such as superDimension™, facilitating the accurate location of lesions and performing biopsies using a position sensor during bronchoscopy within a specific electromagnetic field [12]. ENB has been extensively applied in the biopsy of suspected tumor lesions in the peripheral lung [13]. The National Comprehensive Cancer Network guidelines have recommended using ENB for biopsy in peripheral pulmonary nodule lesions for definitive diagnosis [14]. Previous studies have shown that ENB navigated TBLC can significantly improve the diagnostic yield of small pulmonary nodules with good safety profile [15]. However, there has been only one previous single-arm study of ENB navigated TBLC in the diagnosis of ILD, and the results showed that ENB navigation for TBLC could bring a high diagnostic yield (11/13, 85%) [16]. Therefore, it is necessary to further illuminate the advantages of ENB navigated TBLC in the diagnosis of ILD.

Therefore, this pilot prospectively comparative study was conducted to explore the accuracy of ENB compared to conventional fluoroscopy in TBLC navigation in patients with ILD.

## Material and methods

### Patients

A total of 17 patients participated in the study, all of whom were admitted to the Department of Pulmonary and Critical Care Medicine, China-Japan Friendship Hospital due to ILD between January 1st, 2020 and December 31st, 2022. All patients completed hematological examinations, pulmonary function tests, and high-resolution computed tomography (HRCT) after admission and were evaluated by ILD and respiratory intervention specialists. Further TBLC was recommended by the multidisciplinary team (MDT) to confirm the diagnosis of patients when the following inclusion criteria were met: > 18 years of age; a diagnosis of ILD could not be established after integration of clinical features; forced vital capacity (FVC) > 50%; and diffusing capacity of the lung for carbon monoxide (DLCO) > 35%. Patients who met the following criteria were excluded: acute exacerbation in the past 30 days, bleeding diathesis, anticoagulant therapy, current use of anti-platelet drugs, pulmonary hypertension, respiratory failure, liver or kidney dysfunction, cardiac insufficiency, and platelet count < 50 × 10^9^/L. The site for the lung biopsy is determined through MDT discussion and is typically chosen to target the most prominent lesion site. For diffuse lesions, there is a preference for sampling from the lower lobes. Additionally, the approach includes consideration for sampling multiple segments or lobes, depending on an assessment by an interventional pulmonology specialist. This assessment will evaluate the likelihood of successful sampling and weigh the potential risks involved. This study was approved by the Ethics Committee of the China-Japan Friendship Hospital (2017-25-1), and written informed consent was obtained from all patients.

### Procedures

Patients received either the fluoroscopy navigation group or the ENB group prior to the TBLC procedure were included. TBLC was performed through an endotracheal tube or rigid bronchoscopy under general anesthesia in a hybrid CBCT operation room. For patients in the ENB group. A compact disc of the HRCT scan within 1 week was created for the superDimension™ navigation system (ver. 7.0, Medtronic Inc.).

The cryoprobe positioning of TBLC was divided into two steps. The first step was to determine the initial position of cryoprobe through fluoroscopy or ENB, and then a CBCT scan was performed to confirm whether the cryoprobe position was appropriate. Cryoprobes from ERBE, Solingen, Germany were used in both groups (1.9 mm or 2.4 mm cryoprobe). In the fluoroscopy navigation group, the cryoprobe was advanced as far as possible into the target bronchial segment through the bronchoscopy working channel. Fluoroscopy was then used to navigate the cryoprobe so that the tip of the cryoprobe was 1 to 2 cm from the visceral pleura. In the ENB group, an electromagnetic board was placed on the back of the patient. At first, a portion of the tip of the Extended Working Channel (EWC) would be cut off to allow the 1.9 mm cryoprobe to pass through. A position sensor was then introduced into the airways at the tip of the navigation probe through EWC. With the electromagnetic field generated, the position of the sensor was calculated in real time and merged with the previously created 3D reconstruction of the patient. The sensor was then navigated to the target site in the electromagnetic field according to the predefined biopsy mark. Once the sensor position is determined, remove the sensor and place the cryoprobe through the EWC. After cryoprobe placement by fluoroscopy navigation or ENB, the CBCT imaging (Artis Zee III ceiling, Siemens AG, Munich, Germany) was performed. The cryoprobe position within the lung parenchyma and its relationship to other thoracic structures was assessed using three-dimensional CT images acquired in the axial, coronal, and sagittal planes. If the cryoprobe position was not ideal, it would be adjusted according to CBCT images, and the final biopsy position would be determined, which was generally about 1-2 cm away from visceral pleura.

**Fig. 1:**
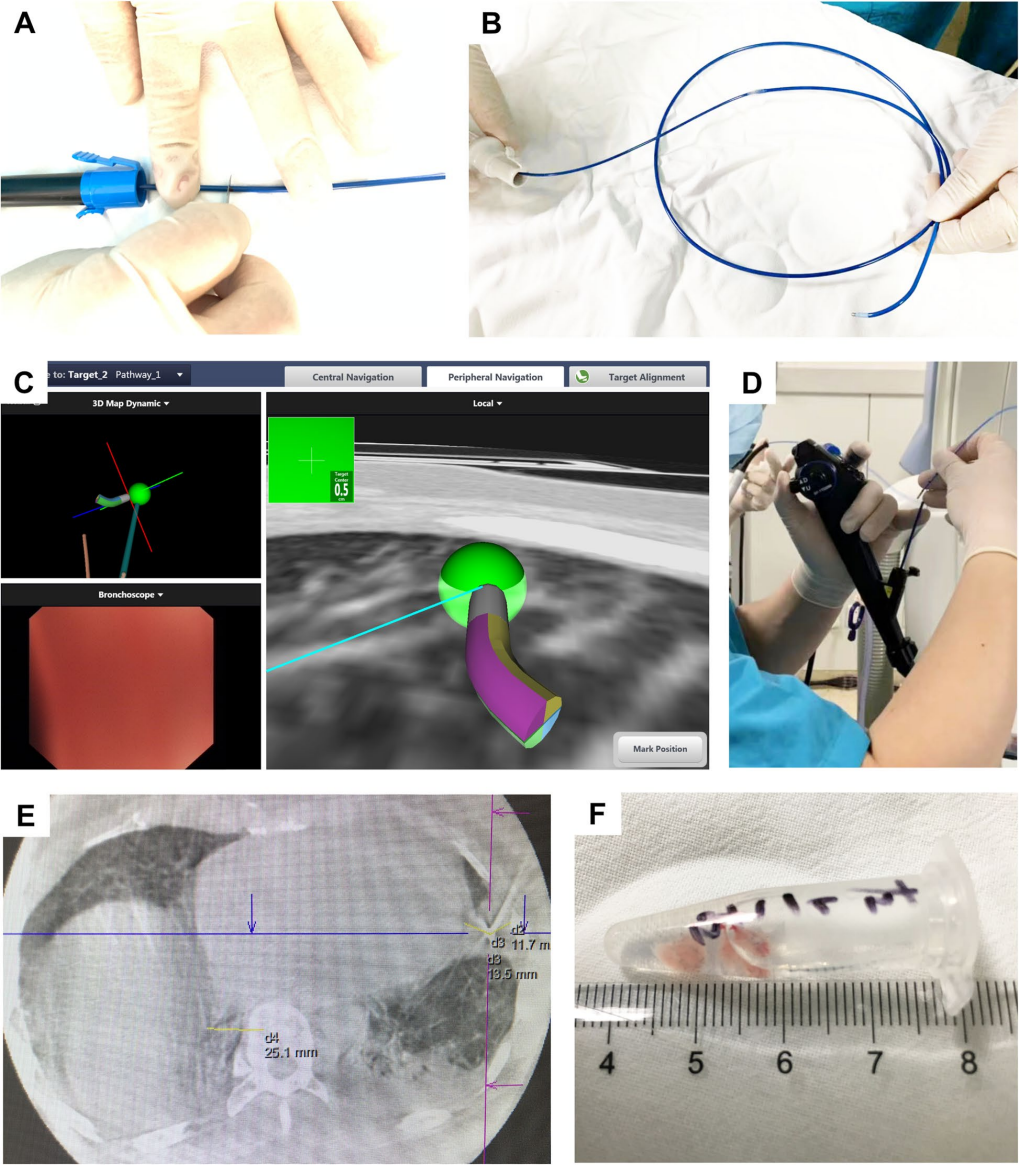
The flow of navigation and TBLC procedure for patients in the ENB group. A portion of the Extended Working Channel (EWC) tip would be cut off to allow passage of the 1.9 mm cryoprobe (**A**). The electromagnetic navigation probe with a position sensor at the tip was placed through EWC (**B**). In the electromagnetic field, used the navigation probe to guide the flexible bronchoscope, and the EWC placed in the working channel to the pre-set biopsy site (**C**). Once navigation is complete, withdraw the navigation probe and position the cryoprobe through the EWC into the target biopsy site (**D**). After cryoprobe placement, CBCT scanning was performed to determine if the cryoprobe position was ideal (**E**). TBLC was performed to obtain lung tissue specimens after confirming that the cryoprobe was in the proper position (**F**)

Cryobiopsy (6–8 s for the 1.9 mm cryoprobe and 4–6 s for the 2.4 mm cryoprobe) was [17] performed after cryoprobe positioning, using carbon dioxide as the cryogen. Pre-positioned bronchial blockers (CRE balloon; Boston Scientific Microvasive, Natick, MA, USA) were immediately filled (0.5–1 atm) after each biopsy to stop bleeding. To check for acute pneumothorax, CBCT imaging or X-rays were operated following the procedure. Four levels of severity were assigned to bleeding [18]: Grade 0, no bleeding; Grade 1, mild bleeding (suction is needed for clearance, but no other endoscopic procedures are required); Grade 2, moderate bleeding (endoscopic procedures such as bronchial occlusion-collapse or ice-cold saline instillations are necessary); Grade 3, severe bleeding (the patient is hemodynamically unstable or has respiratory instability and may need to undergo surgical tamponade or blood transfusions). The flow of TBLC for patients in the ENB group is shown in Fig. 1. TBLC sites, number of cryoprobe adjustments, number and size of specimens, and complications were recorded.

All pathological diagnosis were made by experienced pulmonary pathologists. A second pathologist was consulted in complex cases, and discrepancies were resolved by consensus. The multidisciplinary discussion (MDD) diagnosis was the final clinical diagnosis and was made through discussions among MDT members. There were pulmonary and critical care medicine physicians, radiologists, pathologists, and rheumatologists on the MDT.

### Statistics

Continuous variables were reported as median within the interquartile range (IQR), considering the small sample size of this study. The Mann–Whitney test was used for continuous variables. Categorical variables are presented directly or reported with frequency (percentage). The Fisher exact tests were conducted to compare binary and categorical variables. The statistical analyses were performed with SPSS statistics software (version 25.0; SPSS Inc., Chicago, IL).

## Results

Of the 17 enrolled patients who underwent TBLC, 10 underwent biopsy with fluoroscopy navigation and 7 with ENB. Demographic characteristics, clinical manifestations, and procedure-concerned laboratory tests of the patients are shown in Table 1. There were no significant differences in clinical characteristics between the fluoroscopy and ENB groups.

**Table 1:** Clinical characteristics of patients underwent TBLC

	All (*n* = 17)	Fluoroscopy (*n* = 10)	ENB (*n* = 7)	*P* value
Gender, M/F	8/9	4/6	4/3	0.637
Age, yrs. (Median, IQR)	50.0(43.0–57.5)	47.5(43.2–53.0)	55.0(42.0–60.0)	0.283
Smoking History, Y/N	7/10	4/6	3/4	1.000
Environmental or occupational exposure, Y/N	3/14	3/7	0/7	0.228
Comorbidities, Y/N	6/11	3/7	3/4	0.644
FVC predicted, % (Median, IQR)	86(66–104)	80(62–105)	87(67–103)	0.625
DLco predicted, % (Median, IQR)	67(58–84)	63(52–69)	82(73–86)	0.051
6MWD, m (Median, IQR)	535(503–554)	538(510–563)	520(411–550)	0.354
Minimal SpO_2_ in 6MWT, % (Median, IQR)	92(88–95)	92(85–95)	93(90–95)	0.553
Platelet count, × 10^9^/L (Median, IQR)	207(168–253)	182(166–242)	246(183–273)	0.172
PT, s (Median, IQR)	13.1(12.8–13.5)	13.2(12.9–13.5)	12.8(12.6–13.2)	0.221
aPTT, s (Median, IQR)	36.4(33.7–41.0)	37.0(33.7–42.7)	35.2(31.5–38.3)	0.305

*TBLC* Transbronchial lung cryobiopsy, *ENB* Electromagnetic navigation bronchoscopy, *IQR* Interquartile range, *FVC* Forced vital capacity, *DLco* Carbon monoxide diffusing capacity, *6MWD* Six-minute walk distance, *SpO2* Oxygen saturation of pulse oximetry, *6MWT* Six-minute walk test, *PT* Prothrombin time, *APTT* Partial thromboplastin time

Patients were re-navigated when biopsies were performed in different pulmonary lobes, and a total of 24 navigations were performed in the 17 enrolled patients, 15 navigations in 10 patients using fluoroscopy, and 9 navigations in 7 patients using ENB. The comparison of the biopsy/navigation site, the number of cryoprobe adjustments based on CBCT images, specimen parameters, and procedure-related complications between the two groups is shown in Table 2. There was no significant difference in biopsy/navigation position between the two groups, but cryoprobes in the fluoroscopy group required more adjustments [9/15(60%) v.s. 1/9(11%), *p* = 0.018)] after CBCT confirmed position than those in the ENB group. There was no difference in specimen size and post-procedure complications between the two groups of patients.

**Table 2:** Procedure parameters, biopsy specimens and complications of TBLC

Number of navigations	Fluoroscopy (*n* = 15)	ENB (*n* = 9)	*P* value
Biopsy/Navigation sites, (%)			
RUL	4(27)	1(11)	0.615
RML	2(13)	0(0)	0.511
RLL	4(27)	2(22)	1.000
LUL	2(13)	2(22)	0.615
LLL	3(20)	4(44)	0.356
Times of cryoprobe adjustments, (%)	9(60)	1(11)	0.018
Number of specimens, (Median IQR)	3(1–3)	4(3–4)	0.194
Length diameter of specimen, mm (Median, IQR)	5.3(4.9–5.7)	5.3(4.3–5.8)	0.588
Short diameter of specimen, mm (Median, IQR)	4.0(3.6–4.6)	3.7(2.8–5.0)	0.695
Complications			
Mild bleeding	8(53)	3(33)	0.423
Moderate bleeding	1(7)	0(0)	1.000
Pneumothorax	0(0)	0(0)	–

*TBLC* Transbronchial lung cryobiopsy, *ENB* Electromagnetic navigation bronchoscopy, *RUL* Right upper lobe, *RML* Right middle lobe, *RLL* Right lower lobe, *LUL* Left upper lobe, *LLL* Left lower lobe, *IQR* Interquartile range

The specific imaging findings, pathological and multidisciplinary discussion diagnosis of the two groups are shown in Table 3. A total of 14 patients had a pathological diagnosis, and 15 patients had an MDD diagnosis. Non-specific interstitial pneumonia (NSIP) was the most common imaging manifestation in both groups. NSIP was also the most common pathological diagnosis in both groups. In the fluoroscopy group, three patients could not obtain a definite pathological diagnosis, while in the ENB group, all patients obtained a pathological diagnosis. After MDD, one patient in the fluoroscopy group was diagnosed with unclassifiable interstitial lung disease (uILD), two patients with an indeterminate diagnosis, while all patients in the ENB group had a final clinical diagnosis.

**Table 3:** Imaging performance and diagnosis of patients underwent TBLC

	Fluoroscopy (*n* = 10)	ENB (*n* = 7)
HRCT pattern		
Probable UIP	1	0
NSIP	2	4
NSIP+OP	1	0
OP	1	0
ACIF	1	1
Multiple cysts	1	0
Diffuse micronodules	2	1
Crazy paving	0	1
Miscellaneous	1	0
Pathological diagnosis		
NSIP	3	3
NSIP+OP	1	0
Probable UIP	1	0
RB	2	2
Sarcoidosis	0	1
PAP	0	1
Nondiagnostic	3	0
MDD diagnosis		
iNSIP	0	3
IPF	1	0
RB-ILD	2	2
CTD-ILD	2	0
IPAF	2	0
Sarcoidosis	0	1
PAP	0	1
uILD	1	0
Nondiagnostic	2	0

*TBLC* Transbronchial lung cryobiopsy, *ENB* Electromagnetic navigation bronchoscopy, *HRCT* High-resolution computed tomography, *UIP* Usual interstitial pneumonia, *NSIP* Non-specific interstitial pneumonia, *OP* Organizing pneumonia, *ACIF* Airway-centered interstitial fibrosis, *RB* Respiratory bronchiolitis, *PAP* Pulmonary alveolar proteinosis, *MDD* Multidisciplinary discussion, *iNSIP* Idiopathic non-specific interstitial pneumonia, *IPF* Idiopathic pulmonary fibrosis, *RB-ILD* Respiratory bronchiolitis interstitial lung disease, *CTD-ILD* Connective tissue disease associated interstitial lung disease, *IPAF* Interstitial pneumonia with autoimmune features, *uILD* Unclassifiable interstitial lung disease

## Discussion

TBLC can lead to good pathologic and multidisciplinary discussion diagnostic yield of ILD, but the risk of pneumothorax and airway bleeding is a limiting factor for its application [2–5]. Therefore, accurate navigation is an essential prerequisite for the development of TBLC. Based on the CBCT image as the standard, this study further clarified the application value of ENB in TBLC navigation in patients with ILD by comparing the accuracy of fluoroscopy navigation and ENB.

The biopsy target of TBLC in ILD should not be too close to the pleura or the great vessels to avoid pneumothorax and remarkable airway bleeding [19]. The incidence of pneumothorax in patients with ILD undergoing TBLC without navigation was up to 20.9%, so subsequent studies demonstrated the value of fluoroscopy, radial endobronchial ultrasound (radial-EBUS), CBCT, and ENB in TBLC navigation in this group of patients [3, 16, 20–23]. Given the simplicity and breadth of the application of fluoroscopy equipment, the CHEST Guideline and Expert Panel Report promoted the routine use of fluoroscopy for TBLC in ILD patients [8]. However, whether fluoroscopy navigation could reduce the risk of pneumothorax is still controversial [9, 20, 21]. Radial-EBUS can identify the large vessels in the biopsy field, thus reducing the risk of airway bleeding. Still, the risk of pneumothorax in TBLC using radial-EBUS navigation is high (15%) due to poor judgment of pleural distance [22, 23]. Our team has previously demonstrated the value of CBCT in TBLC through a larger cohort, especially in reducing pneumothorax complications (1.9%) [3]. It is suggested that CBCT navigation may be the safest method for TBLC navigation. However, CBCT has not been widely used in ILD centers. In addition to our team’s previous study, there was only one report on the use of CBCT in TBLC navigation in ILD patients [10], suggesting that accurate and widely used navigation methods should continue to be found.

Many previous studies have confirmed the advantages of ENB in cryobiopsy of peripheral lung lesions, especially pulmonary nodules [13, 24]. It is reasonable to believe the application value of this combination in diagnosing ILD. Kronborg-White et al. conducted a pilot study of ENB as a TBLC navigation tool in diagnosing ILD at multiple European centers [16]. The study used the superDimentation™ system and enrolled 13 patients. With 1.7 mm or 1.9 mm cryoprobe, three patients (23%) developed pneumothorax, one patient (8%) developed mild bleeding, six patients (46%) developed moderate bleeding after TBLC and 11 patients (85%) were finally diagnosed. In our study, the superDimentation™ system was also used. All TBLC in the ENB group used 1.9 mm cryoprobes. With the help of CBCT, no patient developed post-procedure pneumothorax, and there were 11 cases (46% of all navigations) of mild bleeding and 1 case (4% of all navigations) of moderate bleeding. Fourteen of the 17 patients had a pathological diagnosis by TBLC, and 15 had MDD diagnosis, of which 1 had a diagnosis of uILD. The main objective of this study is to observe the accuracy of ENB in TBLC navigation compared with that of fluoroscopy. The results showed that cryoprobe navigation by fluoroscopy required more adjustments than ENB when CBCT images were used as the gold standard for cryoprobe positioning. The results also suggested that ENB has the potential to be comparable to CBCT.

However, ENB presents certain drawbacks in clinical applications. Firstly, ENB is not a truly real-time navigation system; its accuracy is compromised by factors such as patient respiratory movements, localization discrepancies, and short-term variations in pulmonary lesions. To address these issues, patients in this study had completed HRCT within 1 week prior to undergoing TBLC and utilized the most recent imaging for ENB navigation. The majority of ILDs are chronic inflammatory and fibrotic lesions with minimal short-term change. Moreover, CBCT, a true real-time navigation system, was used to calibrate ENB’s accuracy in this study. There was only one cryoprobe adjustment (1 out of 11 biopsies) for TBLC with ENB, suggesting that ENB, despite not being a real-time system, is highly accurate and valuable for further application in diagnosing ILD. If a patient has limited lesions and there is concern that ENB may not accurately access the lesion specimen, the addition of radial-EBUS may be considered to aid navigation. This combined navigation approach has been utilized for biopsy of small peripheral pulmonary nodules and merits further investigation for its value in TBLC for patients with ILD [17, 25, 26]. Secondly, the consumables associated with ENB are expensive, costing more than those for other navigation methods such as fluoroscopy and CBCT. Yet, if ENB reduces the risk of complications associated with biopsy, it may reduce actual healthcare expenditures, so further practical cost-effective studies are needed. Moreover, future developments may introduce more affordable consumables, potentially expanding ENB’s application scenarios. In conclusion, while the current application of ENB in TBLC remains limited, it holds special value in certain scenarios, such as offering a non-radiative option for pregnant patients with ILD who require lung biopsy, which has been previously reported [27, 28]. Pregnant patients were not included in this study, necessitating further research to explore ENB’s potential benefits for this patient group.

There are also some limitations to our study. First, the sample size of the study was very small, which might lead to random error. Although the results showed a significant difference in the accuracy of ENB versus fluoroscopy navigation, the reliability of the results may be low. Second, the decision to use CBCT as the benchmark for navigation accuracy is open to debate, given the scarcity of studies on CBCT’s application in guiding TBLC in ILD patients. Our study’s approach—incorporating CBCT for corrective measures alongside ENB versus fluoroscopy—might have limited our ability to accurately determine the actual complication rates and diagnostic effectiveness of ENB or fluoroscopy-guided TBLC. Future research involving larger cohorts and multi-center trials without CBCT correction for ENB-guided TBLC, is essential to more definitively ascertain ENB’s efficacy in TBLC navigation for ILD patients.

## Conclusions

We performed the first comparison of the accuracy of ENB and fluoroscopy in TBLC navigation in patients with ILD in a prospective pilot trial. When CBCT images were used as reference, TBLC navigation with ENB was significantly more accurate than traditional fluoroscopy. Further studies are still required to explore the application prospect of ENB in TBLC navigation in patients with ILD.

## Data Availability

The data that support the findings of this study are available from Dr. GZ and Dr. HD. These data were used under license for the current study, so these data are not publicly available. However, the data are available from the authors upon reasonable request and with permission from corresponding authors.

## Abbreviations

CBCT: Cone beam computed tomography
DLCO: Diffusing capacity of the lung for carbon monoxide
ENB: Electromagnetic navigation bronchoscopy
EWC: Extended Working Channel
FVC: Forced vital capacity
HRCT: High-resolution computed tomography
IQR: Interquartile range
ILD: Interstitial lung disease
MDD: Multidisciplinary discussion
MDT: Multidisciplinary team
NSIP: Non-specific interstitial pneumonia
radial-EBUS: Radial endobronchial ultrasound
SLB: Surgical lung biopsy
TBLC: Transbronchial lung cryobiopsy
uILD: Unclassifiable interstitial lung disease
